# Comparing the Hierarchy of Keywords in On-Line News Portals

**DOI:** 10.1371/journal.pone.0165728

**Published:** 2016-11-01

**Authors:** Gergely Tibély, David Sousa-Rodrigues, Péter Pollner, Gergely Palla

**Affiliations:** 1 Department of Biological Physics, Eötvös University, Budapest, Hungary; 2 The Design Group, Faculty of Maths, Computing and Technology, The Open University, Walton Hall, Milton Keynes, United Kingdom; 3 MTA-ELTE Statistical and Biological Physics Research Group, Hungarian Academy of Sciences, Budapest, Hungary; Semmelweis University, HUNGARY

## Abstract

Hierarchical organization is prevalent in networks representing a wide range of systems in nature and society. An important example is given by the tag hierarchies extracted from large on-line data repositories such as scientific publication archives, file sharing portals, blogs, on-line news portals, etc. The tagging of the stored objects with informative keywords in such repositories has become very common, and in most cases the tags on a given item are free words chosen by the authors independently. Therefore, the relations among keywords appearing in an on-line data repository are unknown in general. However, in most cases the topics and concepts described by these keywords are forming a latent hierarchy, with the more general topics and categories at the top, and more specialized ones at the bottom. There are several algorithms available for deducing this hierarchy from the statistical features of the keywords. In the present work we apply a recent, co-occurrence-based tag hierarchy extraction method to sets of keywords obtained from four different on-line news portals. The resulting hierarchies show substantial differences not just in the topics rendered as important (being at the top of the hierarchy) or of less interest (categorized low in the hierarchy), but also in the underlying network structure. This reveals discrepancies between the plausible keyword association frameworks in the studied news portals.

## Introduction

Network science has become ubiquitous in describing and modeling a wide range of phenomena in nature and society, applied in studies of systems ranging from the interactions within cells through transportation systems, the Internet and other technological networks to economic networks, collaboration networks and the society [1, 2]. One of the important topics in this area is related to hierarchical organization of networks [3–12], which is mostly motivated by the fact that signs of hierarchy were recorded in numerous real networks. Examples include various animal flocks [13–16], social interactions [17–19], urban planning [20, 21], ecological systems [22, 23] and evolution [24, 25].

A hierarchy is usually depicted as a directed acyclic graph (DAG), where the nodes are layered in different levels, with links pointing from nodes in higher levels towards nodes in lower levels. However, we can distinguish between hierarchies of different types. In a flow hierarchy, the links can represent the chain of command, the flow of information, etc., and in general, they indicate that nodes found in lower levels are influenced by their in-neighbors positioned in higher levels. On the other hand, in a nested hierarchy (also called inclusion hierarchy or containment hierarchy) the links represent an “is a sub-category of” type of relation, as nested hierarchies are obtained by recursively aggregating items into larger and larger groups, resulting in a structure where higher-level groups consist of smaller and more specific components [26]. Prominent examples of nested hierarchy are given by library classification systems and biological classification.

A very interesting problem related to nested hierarchies and classification is given by the automated extraction of nested hierarchies from folksonomies and collaborative tagging systems [27–31]. The association of tags to various on-line contents have became widespread, as various tags may indicate the topic of news-portal feeds and blog post, the genre of films or music records on file sharing portals, or the kind of goods offered in Web stores. These tags usually serve as keywords, providing a rough description of the given entity, helping the users in a fast decision whether the given article, film, etc. is of interest or not. Since the tags appearing in these on-line platforms are usually free words chosen by the author or owner of the given object, they are almost never organized into a pre-defined hierarchy of categories and sub-categories [32–35]. Furthermore, in many tagging systems like Flickr, CiteUlike or Delicious the tagging process is collaborative, as in principle an unlimited number of users can tag photos, Web pages, etc., with free words [36–38]. In order to highlight this collaborative nature, the arising set of free tags and associated objects are often referred to as folksonomies. Since the tagging actions involve user-tag-object triplets, a natural representations of these systems is given by hypergraphs [37, 39–42], where the hyperedges connect more than two nodes together.

In almost all tagging systems the tagged items are allowed to have multiple tags simultaneously, and based on the weighted co-occurrence network between the tags it is possible to extract a nested hierarchy between the tags according to several different algorithms [27–31]. This is usually motivated by the assumption that the way users think about objects or concepts presumably has some built in hierarchy, e.g., “pigeon” is usually considered as a special case of “bird”. Revealing the hidden hierarchy between tags in a folksonomy or in a tagging system in general can significantly help broadening or narrowing the scope of search in the system, give recommendation about yet unvisited objects to the user, or help the categorization of newly appearing objects [42, 43].

Along the above lines, here we apply an improved version of a recent tag hierarchy extraction method [31] to co-occurrence networks between keywords associated to on-line articles, collected from the portals of Spiegel Online, The Guardian, The New York Times and The Australian. The obtained hierarchies show very interesting differences, e.g., the topics rendered as important (being at the top of the hierarchy) in one journal may turn out to be of less interest (categorized lower) in the hierarchy of another journal. In addition, some of the network characteristics of the obtained hierarchies are also quite different, indicating plausible discrepancies in the keyword association frameworks applied in the studied news portals.

## Materials and Methods

### Hierarchy construction

We employ an upgraded version of a recent method [31], which is based on two assumptions:

- tags positioned high in the hierarchy also have high centrality values in the tag-tag coappearance graph,
- parent-child pairs coappear more frequently than expected from pure chance.

According to the first assumption, the algorithm orders the tags by their centrality, then, for each tag (which become child) the parent candidates are collected. All tags with higher centrality are parent candidates of the child tag. Candidate parents are assigned a score, indicating the probability of the observed number of co-occurrences according to a random null-model. Using the second assumption, the final parent is the candidate with the highest score sum, where the sum runs over all descendants of the child tag. Note, that the algorithm builds up the hierarchy bottom up, starting from the leaves with lowest centrality. The full detailed description of the currently used version of the algorithm involving a couple of improvements is given in S1 File.

### Similarity of hierarchies

Hierarchies are frequently represented by Directed Acyclic Graphs (DAGs), in which directed cycles are forbidden. However, children are allowed to have more than one parent in general. For simplicity, we have restricted the number of parents to one in the present analysis. A natural idea for comparing two DAGs is to compare the hierarchical relations, i.e., the sets of ancestor-descendant relationships [31, 44–47]. Here we adopt the approach proposed in Ref. [31], defining a similarity measure based on mutual information. We note that mutual information plays a central role also in the comparison method introduced in [48] for the related, but separate problem of comparing hierarchical community structures, (where only the lowest-level nodes in DAG actually exist in the input data-set). The DAG similarity measure we use, called normalized mutual information (NMI), can be formulated as follows [31]

$$I_{\alpha ,\beta }=\frac{2\sum_{x=1}^{N_{\alpha \beta }}\left|d_{\alpha }\left(x\right)\cap d_{\beta }\left(x\right)\right|\cdot \ln\left(\frac{|d_{\alpha }\left(x\right)\cap d_{\beta }\left(x\right)|\left(N-1\right)}{|d_{\alpha }\left(x\right)|\cdot |d_{\beta }\left(x\right)|}\right)}{\sum_{x=1}^{N_{\alpha }}|d_{\alpha }\left(x\right)|\ln\left(\frac{|d_{\alpha }\left(x\right)}{N-1}\right)+\sum_{x=1}^{N_{\beta }}\left|d_{\beta }\left(x\right)\right|\ln\left(\frac{|d_{\beta }\left(x\right)}{N-1}\right)}$$ (1)

where *α* and *β* are two DAGs, having *N*_*α*_ and *N*_*β*_ tags from which *N*_*αβ*_ are common, *N* = *N*_*α*_ + *N*_*β*_ − *N*_*αβ*_ is the total number of tags, and *d*_*α*_(*x*) is the set of descendants of *x* in DAG *α*. Eq 1 is 0 for independent DAGs and 1 for identical ones. Note that in the strict mathematical sense, Eq 1 is not a mutual information, however, to avoid confusion, we kept the name used in earlier publications.

A further very closely related similarity measure that turned out to be useful in previous studies is given by the linearized mutual information (LMI) [31], based on the fraction of links that have to be rewired in a randomization procedure on *α* leading to a hierarchy *α*_rand_ with the same NMI when compared to *α* as the *I*_*α*,*β*_. The formal definition of this measure is given as follows. Let *I*(*f*) denote the average NMI obtained for a fraction of *f* randomly rewired links, *I*(*f*) = <*I*_original,rand_>_*f*_. By projecting the NMI of the empirical case, *I*_*e*_, to the *f* axis using this function as

$$f^{*}=I^{-1}\left(I_{e}\right),$$ (2)

we receive the fraction of randomly chosen links to be rewired in the empirical case for obtaining a randomized hierarchy with the same NMI. Based on that we define the linearized mutual information, (LMI) as

$$I_{\text{lin}}=1-f^{*}=1-I^{-1}\left(I_{e}\right)$$ (3)

This quality measure corresponds to the fraction of unchanged links in a random link rewiring process, resulting in a hierarchy with the same NMI as the empirical value. (The reason for calling it “linearized is that Eq 3 is actually projecting *I*_*e*_ to the linear 1 − *f* curve). In theory, one might use the minimum ratio of rewired links which gives the same NMI, however, as [31] showed, change in the NMI can strongly depend on the position of the rewired links, therefore, the minimum number can be significantly lower than the average.

### Data

We analyze four tagged datasets, obtained from online news portals. They contain tagged news items, covering a more than 2 years long time window, in the same period. The four sources are: Spiegel Online, The Guardian, The New York Times and The Australian.

#### General observations

There are some observations which hold for all four datasets. For example, very long tags exist, more like headlines (“Muntazer al-Zaidi: the Iraqi shoe thrower”). Some of the tags form frozen cliques in the coappearance network, where each member of such a clique appear only together with the other members of the clique, e.g., “Haiti” and “Haiti Earthquake Disaster 2010”, “Diana” and “Princess of Wales”. Since members of a large clique have large centrality values, such tags will be placed to unwanted high positions by the first step of the hierarchy construction algorithm. Therefore, we have considered such frozen cliques as single tags, which fits better to the assumed usage of tags.

Some concepts are represented by two or more tags, where the same idea is expressed with different, but synonymous words, e.g., “Art” and “Arts”, “The Arab Revolution” and “Arab Spring” or “Japan disaster” and “Japan earthquake and tsunami”. Since the identification of these more complex tags can be done only by a time-dependent context-aware analysis, they were left as observed, unless explicitly stated otherwise. Another problem is posed by the occurrence of very rare tags, that are usually names.

In order to avoid misleading results due to the above observed problems, we have prefiltered the tags by requiring that each tag pair in the coappearance network has to occur on at least *r* news items. The *r* = 1 case corresponds to skipping the prefiltering. We set *r* to its optimal value for each dataset by keeping the number of tags as high as possible and minimizing the number of misleading tags described above. Finally we note, that temporarily important topics can produce unexpected co-occurrences (e.g., “Japan” -> “Fukushima Nuclear Catastrophe” -> “Nuclear Power”).

#### Spiegel Online

The dataset is from April 2011 to January 2013. It contains 4802 news items and 388 tags. For the pre-filtering, minimum 1 common news item for each tag pair (i.e., no filtering) seems to be a good trade-off between noise reduction and info loss. The dataset looks very well organized (e.g., there are only 400 tags, general tags are used consistently, and there are only a few duplicated tags, long tags or frozen cliques).

#### The Guardian

The dataset is from November 2009 to January 2013, containing 55835 news items and 6797 tags. Pre-filtering needs minimum 3 news items (removes 2530 tags and 61 news items). Here we found several ad hoc tags (mostly names), that were used only once or a handful of times. We found synonymous tags, e.g., “Middle East and North Africa” and “Middle East”, that will appear as two local roots of two branches in the DAG. These branches correspond to the same topic, thus, divide the related tags between them.

#### The New York Times

The dataset reaches from November 2010 to January 2013. It contains 35736 news items and 23009 tags. Cliques are a huge problem here. There are 2902 ones, collapsing them removes about 6000 tags. Several cliques appear on numerous objects, therefore, the minimum news item-filtering does not solve the problem automatically. Cliques also reach very large sizes: there is a 809-tag clique (may contain much more characters than a news item itself); after the minimum news items filtering, the largest one still consists of 44 tags—as follows from the definition of cliques, these tags appear strictly together on each object. For the pre-filtering, minimum 5 news items were required, leaving finally 2981 tags (out of 23009). News items were much less affected, 31184 out of 35736 remained.

#### The Australian

Data is from December 2009 to January 2013. It contains 31501 news items and 79054 tags—thus, there are much more tags than news items. Cliques are present, but have only 1–2 objects, so it is not a serious problem, the pre-filtering can solve it. Multiple synonyms occur on the same object very often—e.g., “Economist_Paul_Samuelson” “Paul_A._Samuelson” “Paul_Samuelson”. Another example is the set of synonyms for Barack Obama, which are: “Barack_Obama”, “BARACK_Obama”, “Obama”, “PRESIDENT_Barack_Obama”, “President_Barack_Obama”, “President_Obama”, “US_PRESIDENT_Barack_Obama”, “US_President_Barack”, “US_President_Barack_Obama”, “barack_obama”. Pre-filtering with minimum 5 news items leaves 1673 tags out of 79504. The news items are reduced from 31501 to 10550. The tags have relatively few objects, and not only due to the large number of very infrequent tags, e.g., even the prime minister has only 900 objects. Although there are very general tags like “community”, “committee” or “claim”, most of tags are very specific, almost tailored for one object, e.g., “rebels_storm_Gaddafi_compound”.

## Results

We analyzed the tag hierarchies obtained from an improved version of “algorithm B” published in Ref. [31]; a brief description of the idea of the method can be found in Materials and Methods, the full details of the used algorithm are given in the S1 File. In Analysis of the individual tag hierarchies first we summarize the most important properties of the individual hierarchies corresponding to the different news portals, which is followed by the pairwise comparisons in Pairwise comparisons. Finally, in Statistical properties of the overall hierarchy structures we examine the overall quality of the hierarchies from different aspects.

### Analysis of the individual tag hierarchies

#### Spiegel Online

**Fig 1:**
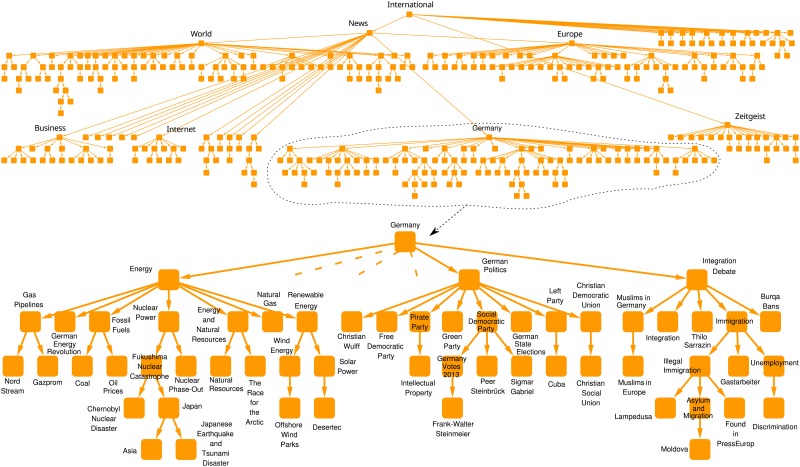
Overview of the Spiegel DAG (top), and one part enlarged (bottom). The DAG is broken into two lines in the top figure to fit the whole graph in the available width. On the bottom figure, dashed links indicate descendants of the tag “Germany” which are not shown.

The constructed DAG consists of 1 connected component. Most of the tags are under 3 branches: “World”, “Europe”, “Germany”. A visualization of the DAG is shown on Fig 1. The Spiegel DAG seems to be somewhat concerned with immigrants and integration, they have a branch containing 3.9% of the tags, similarly to Australian’s 4.4%, and in contrast to 0.1% and 0.7% of Guardian and NYT (note that the latest data come from January 2013, well before the beginning of the recent migrant crisis).

#### The Guardian

**Fig 2:**
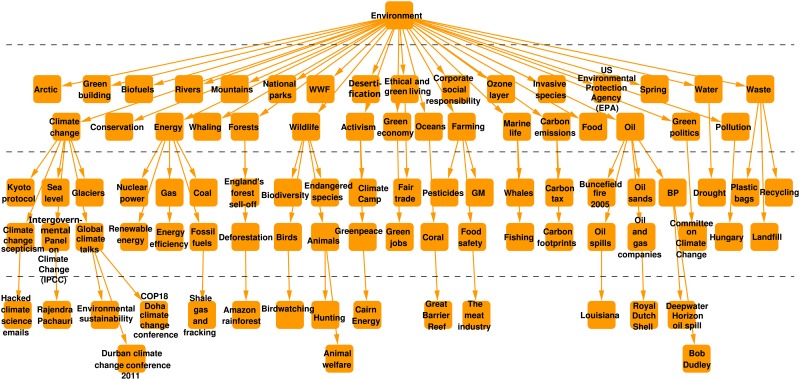
Part of the Guardian’s “Environment” branch, in the component “World news”. Hierarchical levels are separated by dashed lines.

The overall structure of the DAG is quite well organized, the top 2–3 levels are very impressive. The DAG consists of four similarly-sized connected components: “UK news”, “World news”, “Culture”, “Sport”, although the tags “World news” and “UK news” are in isolated components, they are not completely mutually exclusive, e.g., both of them appear on the news items of “Defence policy”. Note that while the components’ top tags correspond well to the menu items on the journal’s website, they are placed totally automatically by the DAG construction algorithm. Visualization is omitted due to the relatively large size of the DAG, however, a smaller sample is shown on Fig 2.

#### The New York Times

Here we found numerous duplicated branches in the constructed DAG (e.g., for research, television, education, medicine, defense and military forces). This indicates that for these topics, two distinct sets of tags were used in parallel. The DAG is much less organized than that of the Spiegel and of the Guardian. There are 31 isolated components, most of them correspond to one theme (e.g. “Baseball”). The sizes of the components varies from 898 to 2, and there is a continuous range of them from the 2nd largest one (274 tags) down. There are no very general categories. Although a number of large related components exists (under the tags “Basketball”, “Baseball”, “Football”), these components are not collected under a general “Sport” tag. It seems as if there were no demand for using general tags. Note that there is a tag called “sports”, however, it appears only on 5 news items, and it is negligible. A technical consequence is that the DAG construction algorithm does not always select the most general tags as roots, because they lack the important connections to other components. Instead, one of the more specific tags can be selected for a central position, for example, “Middle East and North Africa Unrest (2010-)” for foreign affairs, or “European Sovereign Debt Crisis (2010-)” for Europe-related tags. In other words, the centrality no longer correlates only with the generality for the top tags. Some lower-level branches end up at unexpected places, e.g., “Environment” under “Iran”. Superfluous levels appears, for example, “International Relations” under “United States International Relations”.

#### The Australian

The DAG looks disorganized overall. There are about 1900 components for the 79504 tags without the pre-filtering, and about 300 components for the min. 5 news items-filtered 1673. There are no macroscopic components, the largest one’s size is just 3480 (out of 79504 tags) and 165 (out of 1673 tags), which is less than 10% of the total nodes. Even the existing components look more like just bunches of more or less associated tags than small hierarchical structures.

In general, the top of the constructed DAGs are much better than the bottom. This is no surprise—there is much more information for the construction algorithm at the top of the DAG.

### Pairwise comparisons

We carried out a pairwise comparison between the journals from the point of view of their content organization. Since the audience and the interests of the journals are different, the list of tags appearing on the articles was unique for each news portal. Therefore, before actually comparing the tag hierarchies, first we needed to create a common tag set for each pair of journals. In a number of cases, finding the corresponding tag pairs went beyond a simple string matching and was based on semantic matching, e.g., “Fossil fuels” (Guardian) was matched with “Oil (Petroleum) and Gasoline” (NYT). Matching was done manually, which may limit the analysis of datasets larger than the ones presented here. The size of the reduced common tag sets were 252 (Spiegel-Guardian), 217 (Spiegel-NYT), 985 (Guardian-NYT), 93 (Australian-Spiegel), 278 (Australian-Guardian), 274 (Australian-NYT).

The reduced hierarchies were obtained by keeping only the common tags in the original DAGs and erasing the rest of the tags. In most cases this resulted in deletion of leafs, sub-branches, or lower parts of sub-branches from the original hierarchies. However, a small number of times this procedure erased a tag higher in a given branch while keeping other tags lower in the same branch, therefore, distorting the original DAG structure in a radical way. To ensure as much similarity to the original hierarchies as possible, under these circumstances the ancestors standing higher in the branch were also kept, despite that they were not part of the common tag set, (see S1 File for more details). The reduced DAGs can be found in S1 File.

**Fig 3:**
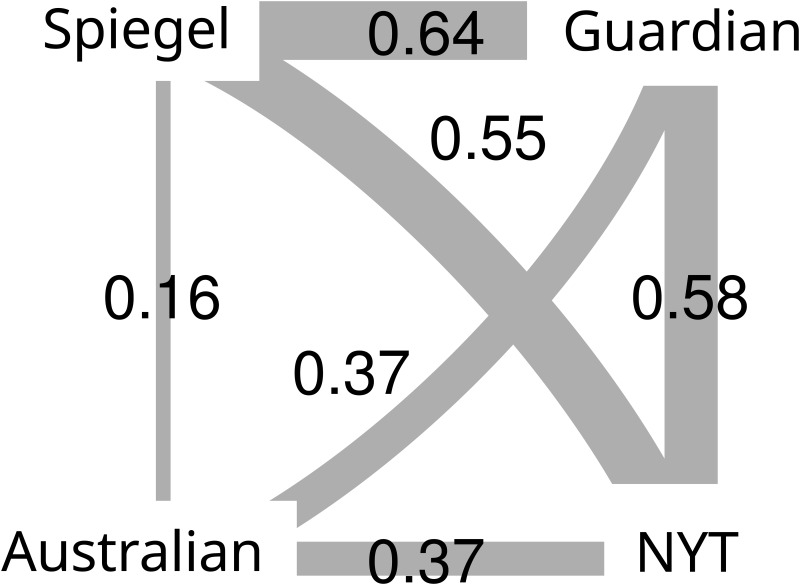
Similarities between the news portals’ DAGs, according to the mutual information-based linearized information similarity measure described in Similarity of hierarchies.

**Fig 4:**
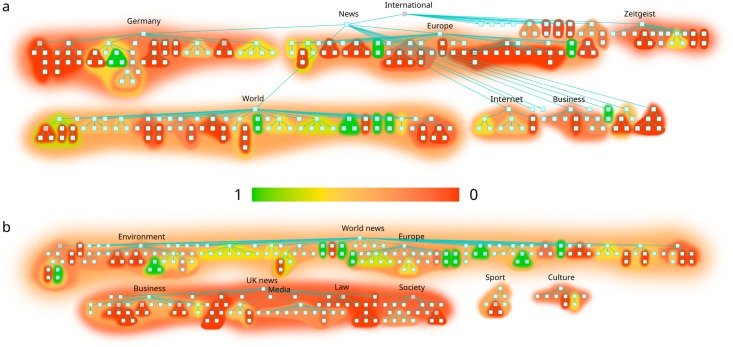
The Spiegel (top) and the Guardian’s (bottom) reduced DAG structures, providing the largest overall similarity in our analysis. For clarity, Spiegel’s DAG is broken into two lines. Background colors show the result obtained by applying the similarity measure given in Eq 1 to the given branch and the most similar branch from the other hierarchy. Note that sub-branches on all hierarchical levels have their own color.

For each pair of journals we have computed the linearized information similarity measure described in Similarity of hierarchies between the reduced DAGs, the obtained values are shown in Fig 3. According to the results Spiegel and Guardian provide the largest similarity measure, which is also supported by a number of identical or almost identical sub-branches between the two DAGs, as shown in Fig 4. Here the background coloring of the sub-branches indicate the similarity to the corresponding (most similar) sub-branch in the other DAG.

The Spiegel, the Guardian and the New York Times have an overall similar structure, as Fig 3 shows, opposed to the Australian, which is dissimilar to all of them. Still, there are some differences between the first three journals. The Guardian, compared to the Spiegel, has a level of intermediately-sized branches, e.g., “law” or “society” in “UK news”. This level is missing from the DAG of Spiegel. Their global DAG structures are shown in Fig 4.

**Fig 5:**
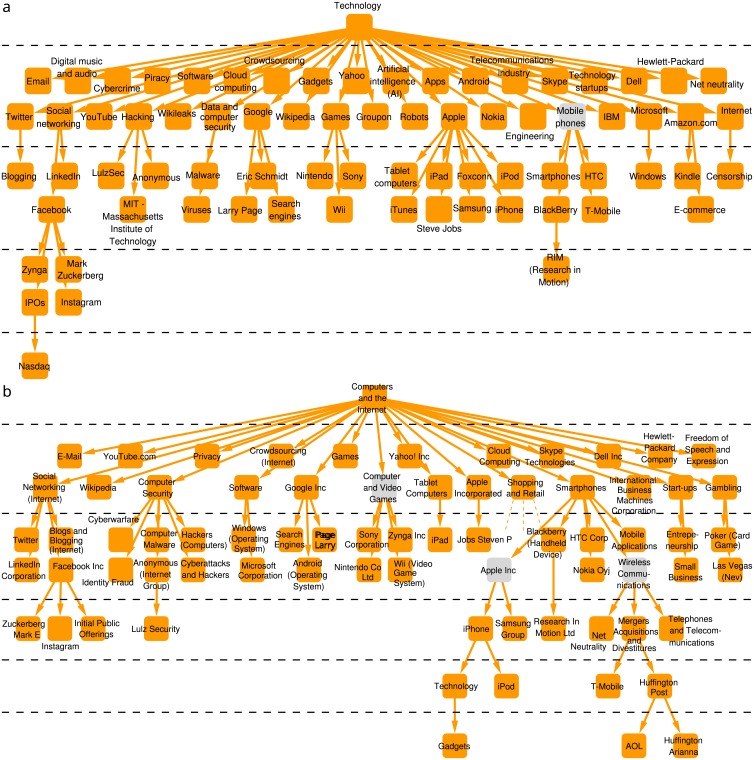
Guardian’s “Technology” branch (top) and New York Times’ “Computers and the Internet” component (bottom). Hierarchical levels are separated by dashed lines. Grey tags do not appear in both DAGs, however, they connect branches containing common tags.

Meanwhile, the New York Times has interestingly no “World” tag, and foreign countries are separated into 4 different branches, in 3 components (see S1 File for more details). Although the linearized information similarity between the Guardian and the New York Times is somewhat lower, they also have a few quite similar branches; a prominent example is shown in Fig 5.

### Statistical properties of the overall hierarchy structures

According to the results presented in the previous sections the tag hierarchies obtained for the studied journals show strong differences. Here we examine to what extent does their overall structure follow a few simple intuitive requirements that can be formulated for a well organized tag hierarchy.

#### Correlations with Google News

One of the basic properties of a well organized hierarchy is that frequent, more general tags are expected to be higher compared to rare, specific tags. In order to examine the obtained hierarchies from this perspective we compared the centrality score of the tags in the tag co-occurrence network (determining their position in the hierarchy) with their number of hits provided by Google News. For each pair of tags with a significant number of co-occurrence we checked whether the difference between their centrality score and the difference between their number of hits in Google News have the same or the opposite sign. If the signs of the differences match for the majority of the tag pairs, then we can assume that the structure of the hierarchy is consistent with word frequencies of English news texts around the world. Although the magnitudes of the differences are not compared, large relative deviations can also mean that the signs change in other relations of the affected tags, thus, tracking the signs can reveal large relative deviations in certain cases.

In Table 1 we show the relative frequency of the cases, where the differences have the opposite sign, calculated for tag pairs co-appearing in statistically significant numbers. If tags are assigned to articles absolutely at random, the result would correspond to a 0.5 inversion rate, i.e., half of the coappearing tag pairs would have similar centrality and frequency ordering. According to Table 1, the Spiegel and the Guardian data sets provide the best correspondence between tag frequency and centrality, with only a few percent difference in their score. They are followed by the New York Times, and finally, the Australian has a score close to the random case. Although the Google News data may be somewhat different from a fictitious collection word usage of all English speaking journalist, the results in Table 1 show a quite clear-cut picture, which also corresponds well to the results of other comparisons.

**Table 1:** Ratios of inversions between centralities and real-world occurrence frequencies, calculated for tag pairs coappearing in statistically significant numbers. Totally random case corresponds to 0.5.

dataset	ratio of inversions
Spiegel	0.19
Guardian	0.21
New York Times	0.31
Australian	0.44

#### Geometrical properties of the hierarchies

In this section we focus on the geometrical properties of the tag hierarchies from the perspective of whether their structure is helping navigability and search. First we examine the fragmentation of the DAGs, which we can quantify by first introducing the average size of the component of a randomly chosen tag given by,

$$\tilde{s}=\frac{\sum_{i}^{\text{tags}}s_{i}}{N}$$ (4)

where *s*_*i*_ is the size of the component containing tag *i* and *N* is the total number of tags. Based on $\tilde{s}$ we can calculate the expected lowest hierarchy level *l* on which the top node of a branch of size $\tilde{s}$ would appear in a balanced *k*-ary tree of size *N*. In such a tree any branch can contain at most half of the tags of its mother branch, thus, we define *l* as

$$\begin{array}{cc} l & =\left\lceil \log_{2}N/\tilde{s}\right\rceil ,\;\tilde{s}<N\\ l & =1,\;\;\;\;\;\;\tilde{s}=N \end{array}$$ (5)

where ⌈*x*⌉ denotes the ceiling function of *x*. The value of *l* becomes high for strongly fragmented tag hierarchies consisting of many small isolated components, where the navigability of the hierarchy is low. The results for $\tilde{s}$ and *l* are summarized in Table 2. The tag hierarchy obtained for Spiegel (consisting of a single component) provides the lowest *l* value, followed by Guardian and New York times. Apparently, the DAG of Australian is showing a very fragmented structure with *l* = 6.

**Table 2:** Characteristic level showing the highest level of an idealized hierarchy to which an average connected component corresponds.

dataset	$\tilde{s}$	*N*	*l*
Spiegel	388	388	1
Guardian	1338.7	4263	2
New York Times	384.2	2945	3
Australian	46.2	1487	6

Another important question is whether branch sizes are balanced or not in the hierarchies. A well-balanced hierarchy is expected to have at least 2 but not more than O(1) comparably sized branches at every nonleaf tag. We define a balancedness measure with a pair of real numbers from [0, 1) × [0, 1) corresponding to the ratio of “giant branches” and the ratio of “dwarf branches” in order to quantify how a DAG fits to the above criterion. First, we calculate the cumulated size of the branches having a child branch which contains more than 50% of the parent branch’s tags. Second, we calculate the cumulated size of the child branches which are smaller than 10% of their parent branches. The higher threshold is motivated by the fact that a child branch above 50% is larger than all the other child branches combined. The motivation for the lower threshold is that below 10%, for equal-sized child branches, the number of child branches exceeds O(1). Other numerical threshold values might also be applied, however, for demonstrating significant phenomena the precise value of the thresholds should not be important. We normalize the sums by their maximal possible value, thus, our balancedness measure is given by

$$\left(R_{g},R_{d}\right)=\left(\frac{\sum_{g}S_{g}}{\sum_{b}S_{b}},\frac{\sum_{d}S_{d}}{\sum_{b}S_{b}}\right)$$ (6)

where *b* goes over all branches containing at least 2 tags, *S*_*b*_ is the size of branch *b*, *g* goes over branches containing a sub-branch having more than 50% of *g*’s tags, and *S*_*g*_ is the corresponding branch size, and *d* goes over sub-branches which are smaller than 10% of their parent branches with *S*_*d*_ being the corresponding branch size. A perfectly balanced hierarchy would have a (0, 0) score and the two extremely unbalanced cases would have (1, 0) for a chain and (0, 1) for a star graph. The results for (*R*_*g*_, *R*_*d*_) are given in Table 3.

**Table 3:** Ratios of giant and dwarf branches among all branches, size-weighted.

dataset	*R*_*g*_	*R*_*d*_
Spiegel	0.32	0.22
Guardian	0.10	0.42
New York Times	0.42	0.22
Australian	0.26	0.17

Spiegel’s *R*_*g*_ is dominated by a single contribution. The global root, “International” has a branch containing almost the whole DAG under “News”. Most of *R*_*d*_ comes from small branches, although there are a few exceptions. In the Guardian DAG, dwarf sub-branches are common, due to the huge size of the components which dwarf several branches, as well as to nearly star-shaped branches, sometimes containing hundreds of leaf-tags (e.g., “Film”, “Music”). For the NYT, contrary to the Guardian, *R*_*g*_ is much larger than *R*_*d*_. Two important reasons are misplacing a number of branches and letting less general tags getting high centralities. Since the Australian DAG has quite limited structure inside the numerous small components, *R*_*g*_ and *R*_*d*_ are not very informative measures here. However, the tiny components seem to be well balanced.

Further analysis of the DAGs can be found in S1 File.

## Discussion

We studied the hierarchy of keywords associated to news articles in four different on-line news portals. The datasets contain various artifacts, such as long and complex keywords, frozen cliques of exclusively coappearing tags, synonyms or very rare and specific tags. Nonetheless, it was possible for the construction method to obtain very reasonable DAGs from the data. The identification of frozen cliques might also be applied by disambiguation techniques, to identify cliques of equivalent semantic meaning, used in the field of Natural Language Processing. The constructed DAGs suggest that the tags appearing in the different news portals are organized to different degrees. Our analysis revealed that Guardian has an extra intermediate level of organization at certain locations. A further very interesting result is that the number of connected components in the DAGs conveys information about the extent of organization in the data: the Spiegel and Guardian have O(1) components and are quite organized, the New York Times has a few dozen components and breaks the world into independent pieces, and the Australian has O(100) components which are barely informative at all.

A similar picture was emerging from the comparison between the frequencies of tags in Google News and their centrality score in the tag-tag co-appearance graphs. The correlation was quite strong in case of the Spiegel and the Guardian, medium for the New York Times, and almost equivalent to the totally random case for the Australian. A more detailed characterization of the DAGs can be obtained by quantifying the extents of too large and too small sub-branches. Although being a geometry-based analysis, it can also identify problems with tag functions, like a non-comprehensive set of intermediate-level branches in the Guardian, or misplaced branches in the New York Times.

In summary, the following picture is arising from the different analyses we carried out: the Spiegel and Guardian datasets are quite well-organized, the New York Times is significantly less but still has relevant hierarchical structure, and the Australian is close to being random, from a hierarchical point of view. The consistency of the results is encouraging, and suggests that the measures used are useful in the quantification and comparison of datasets from the aspect of hierarchical organization.

## Supporting Information

S1 File S1 File provides more details on the applied algorithm and on the pairwise comparisons of the DAGs. (PDF)
