# Supplementary material for: Comparing the Hierarchy of Keywords in On-Line News Portals
Source: PLoS One. 2016 Nov 1;11(11):e0165728. doi: 10.1371/journal.pone.0165728 (PMC5089747; doi:10.1371/journal.pone.0165728)
Supplement: S1 File — (PDF) [file pone.0165728.s001.pdf]

Supplementary Information

Comparing the hierarchy of keywords in on-line news portals

Gergely Tibély, David Sousa-Rodrigues, Péter Pollner and Gergely Palla

Construction algorithm updates

We updated the DAG construction algorithm of [1] with the following features:

- if a candidate parent gets most of its votes only from a small fraction of the descendants, then its score is reduced. However, "small fraction" is calculated in a weighted manner: a direct child counts with a weight of 1, a grandchild with a weight of e^{-1}, etc. a descendant 10 levels below with e^{-10} weight. It is also good for preventing a misplaced branch at the bottom of another branch to distort the upper branch's parent selection process. Note that the votes of the descendants (z-score or other) remain unchanged.
- z-score is used only for selecting the statistically significant coappearances. For parent selection we introduced a new score, defined as

$$z^* = \frac{\#\text{common objects} - \text{expected value}}{\ln(\#\text{objects of candidate parent} + 10)} \tag{1}$$

  Expected value is the same as for the z-score. Here we replaced the linear normalization with logarithmic one in order to prevent very frequent tags to become the immediate parent of almost all tags. More generally, it manifests the principle that the parent should be the most specific tag among the ancestors. The logarithmic scaling replaces the simple linear scaling, which is too strong. The +10 factor is to hold back the very infrequent candidates.

Overview of the DAG construction algorithm

The DAG construction method relies on two assumptions: tags high in the hierarchy are more central in the tag-tag cooccurrence graph, and that tags close to each other in the DAG have significantly high cooccurrences. Thus, the algorithm first calculates tag centralities in the tag-tag cooccurrence graph, then tries to find a parent for each tag among those having a significantly high cooccurrence with the current tag.
In detail,

1. Input is read. Among the tag-tag cooccurrence links, those are kept which reach the predefined minimal threshold.

2. Z-scores of the links are calculated. The new z^* score, defined by Supplementary Equation 1, is also calculated. Z-score or z^* is set to 0, if one of the tags, or the "candidate parent" tag appears on all objects, respectively.

3. Eigenvector centralities of the tag-tag coappearance graph are calculated:

   (a) only links above a preset z-score threshold are taken into account, except when one of the tags appear on at least half of the objects of the other tag. The link weights are taken into account.

   (b) the eigenvector centralities are calculated by the power iteration method, initialisation is done according to the strengths (sums of link weights) of the tags.

   (c) normalisation during the iteration is done for each connected component separately.

   (d) a teleportation factor is applied; default teleportation probability is 0.15, which is a widely used value. We apply link teleportation [2], i.e., target tag probabilities are proportional to the tag strengths.

   (e) the number of iterations is 100.

   (f) if the difference between the vectors in the last two iteration steps is not sufficiently small, suggesting that the vector is oscillating, i.e., the graph is near to a bipartite graph, the average of the last two vectors is taken, which solves the problem of oscillation.

   (g) if the graph for which the centrality calculation was done consisted of more than one connected component, a second eigenvector centrality is also calculated on an extended graph. Extension means including all links between the connected components, regardless of the z-scores. The aim is to have comparable centrality values when considering a parent-child pair which are in different components.

4. DAG construction:

   (a) for each tag, a parent tag is looked for. Tags are considered in ascending order of centrality, i.e., the DAG is built bottom-up. If multiple parents are asked for, the first step is still to find a single one by applying the method below.

   (b) for each candidate parent, we calculate a score. It consists of the sum of the z^* values between the candidate and the already found *descendants* of the current tag, plus the z^* of the candidate and the current tag itself. The candidate having the highest score is chosen as parent. Conditions:

      i. the candidate should have a higher centrality value than the current tag. If the current tag belongs to a component smaller than 5 tags, the single component-centrality is used.

ii. there should be above z-score threshold links between the candidate and the voting descendants, and as well as the current tag. Exceptions as described earlier are accepted.

(c) if we enforce a fully connected DAG, i.e., which consists of one connected component, and there is no valid candidate parent for a tag, then the parent finding procedure is reapplied, without the z-score criterion. Accordingly, the single component centralities are used. Also, in these cases having poor statistical qualities, instead of the z^* values, the simple cooccurrence numbers are summed in the candidate scores. At least one cooccurrence with the current tag is still required.

(d) in order to avoid parents elected by only a small minority of the descendants, the candidate scores are multiplied by a factor depending on the number of descendants contributing to the score of the candidate:

i. this reduction is not applied if the candidate was selected in the second, z-score thresholdless run. In that case, any candidate satisfies.

ii. the reduction is also avoided if the number of descendants is less than 5.

iii. the descendants of the current tag are counted, but descendants being farther from the current tag contribute less to the count than those being closer. Descendants far below the current tag are, on the one hand, not expected to have links to candidate parents, on the other hand, tend to be numerous and therefore distort the statistics. Quantitatively, contribution is $\exp(-(\text{number of levels from the current tag}))$. The sum is denoted by N.

iv. do the same for the descendants contributing to the score of the candidate parent. The sum is denoted by n.

v. the candidate score is multiplied by a mirrored Fermi-function, i.e., it is very close to 0 at 0, very close to 1 at 1, and follows a softened step-like curve (Supplementary Fig. S1):

$$r = \frac{1}{1 + e^{-50\cdot(\frac{n}{N} - 0.15)}} \tag{2}$$

The parameter values are based on empirical data. The step from 0 to 1 is at 0.15, 50 sets the softness of the step. Note that the argument of the function, n/N, is always between 0 and 1, independently from the DAG. One might observe that in case of the minimal 5 descendants, $1/5 > 0.15$, so calculating the correction looks irrelevant. Note, however, that due to the softness of the step function, the correction factor is still lower than 1 in this case.

vi. the z^* between the candidate parent and the current tag is added to the score only after the reduction by Supplementary Equation 2.

(e) if there is a tie in the first place of the candidate score, the candidate having the higher single component centrality (i.e., the more general tag) is chosen. Centralities are real numbers, ties among them are not expected.

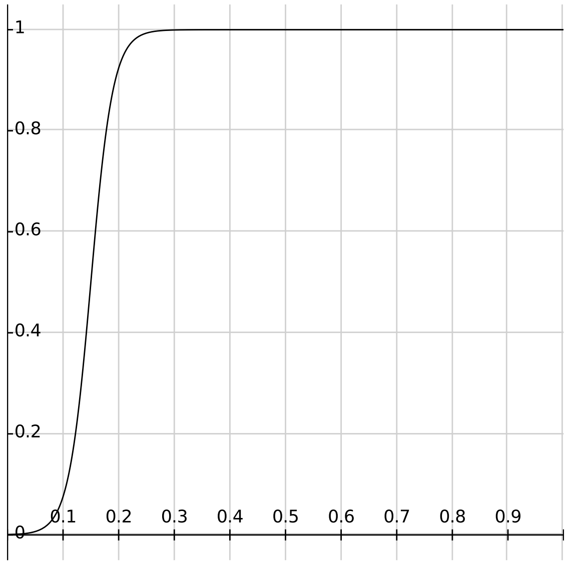

Supplementary Figure S1: The mirrored Fermi-function.

(f) if multiple parents are allowed, all candidates are accepted as parents which have at least as high z^* with the current tag as the first parent. Here, descendants are not taken into account.

(g) if a tag was able to get a parent only by omitting the z-score threshold, no multiple parents are looked for.

Phantom nodes

During the pairwise DAG comparisons, in certain situations, tags not appearing in the other datasets are retained. These occure when a noncommon tag have at least two subbranches containing common tags (see Supplementary Fig. S2). Such tags are omitted in the similarity calculations, but their branches are taken into account.

Pairwise comparisons of the DAGs

On Supplementary Figs. S3-S14, the reduced DAGs with tag names are shown, without singleton components.

An interesting observation is that the NYT DAG does not have a `World` tag. The most comparable tag is `United States International Relations`. Foreign countries, however, appear altogether in 4 different branches, in 3 components: two corresponding to the Middle East (1 for countries of military concern for the US, 1 for the others), one for those primarily having economical significance, and one for Europe. The names of the corresponding local roots are `European Sovereign Debt Crisis`, `Economic Conditions and Trends` under `United States Economy`, `United States International`

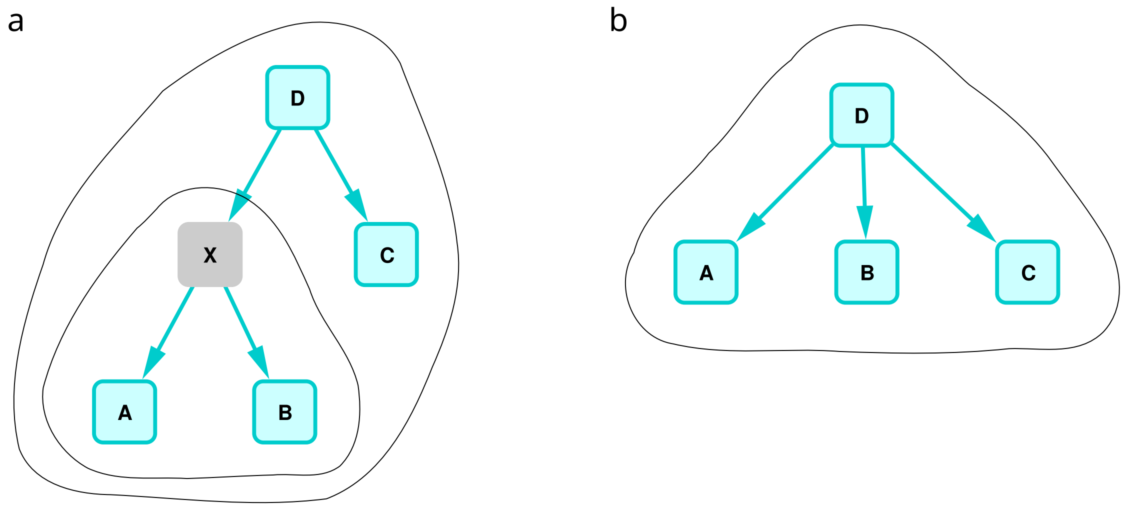

Supplementary Figure S2: An example where including or removing a tag ("`X`", grey) results in different branch structures. Branches are highlighted by outlines.

`Relations` for the military-associated branch, and `Middle East and North Africa Unrest`.

Due to the widespread use of synonyms, only for the Australian dataset, synonyms in the pairwise reduced DAGs were collapsed. 69-225 tags remained after the reduction to the common tag set. The DAGs have several ($\mathcal{O}(10)$) very small components, the largest one containing 15 tags. Note that the corresponding reduced DAGs of Spiegel, Guardian and New York Times retain their original component structure and do not fragment.

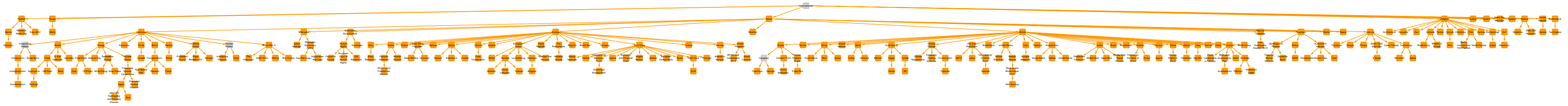

Supplementary Figure S3: Spiegel, reduced for Guardian. Use Ctrl-F to search for specific tags.

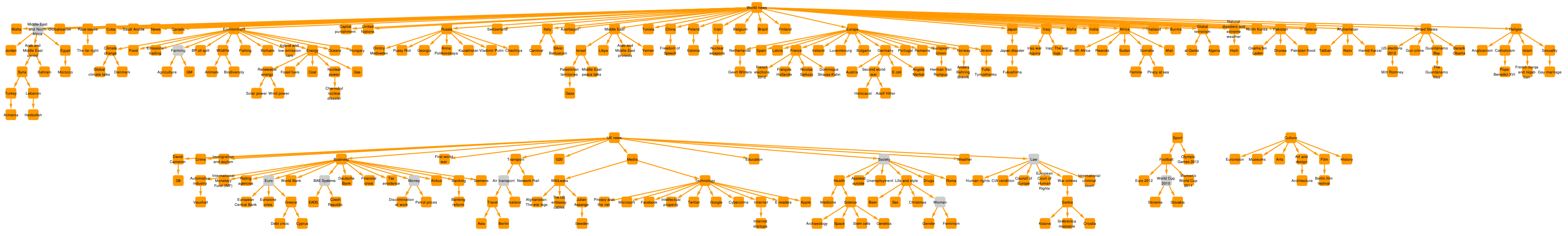

Supplementary Figure S4: Guardian, reduced for Spiegel. Use Ctrl-F to search for specific tags.

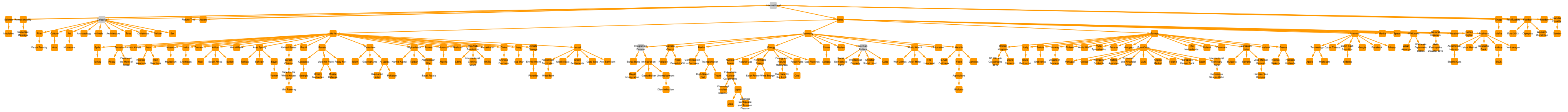

Supplementary Figure S5: Spiegel, reduced for New York Times. Use Ctrl-F to search for specific tags.

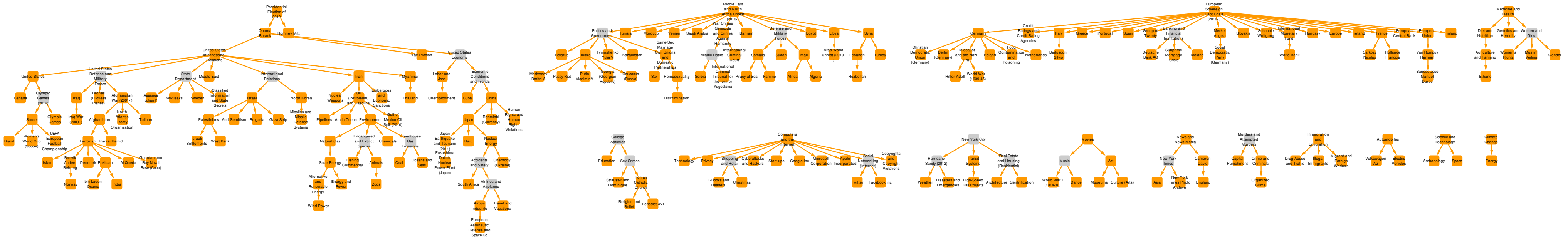

Supplementary Figure S6: New York Times, reduced for Spiegel. Use Ctrl-F to search for specific tags.

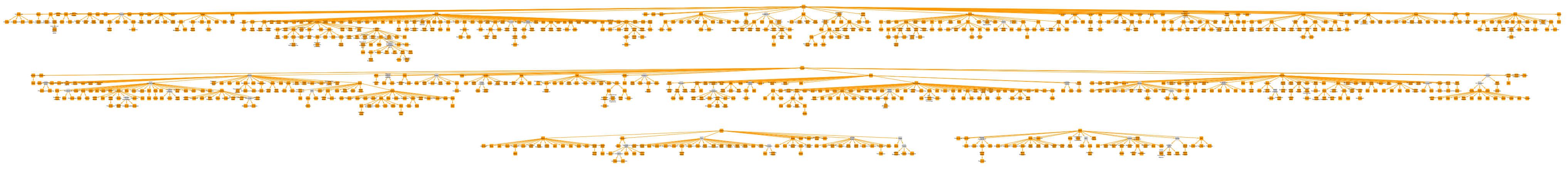

Supplementary Figure S7: Guardian, reduced for New York Times. Use Ctrl-F to search for specific tags.

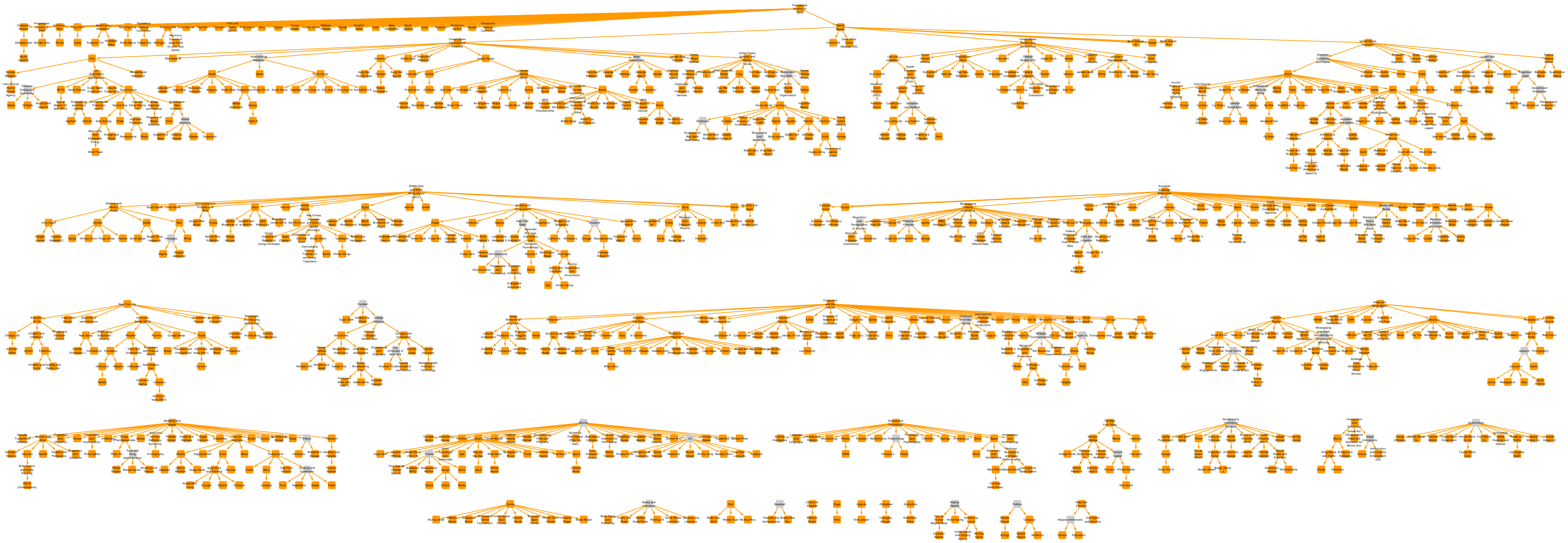

Supplementary Figure S8: New York Times, reduced for Guardian. Use Ctrl-F to search for specific tags.

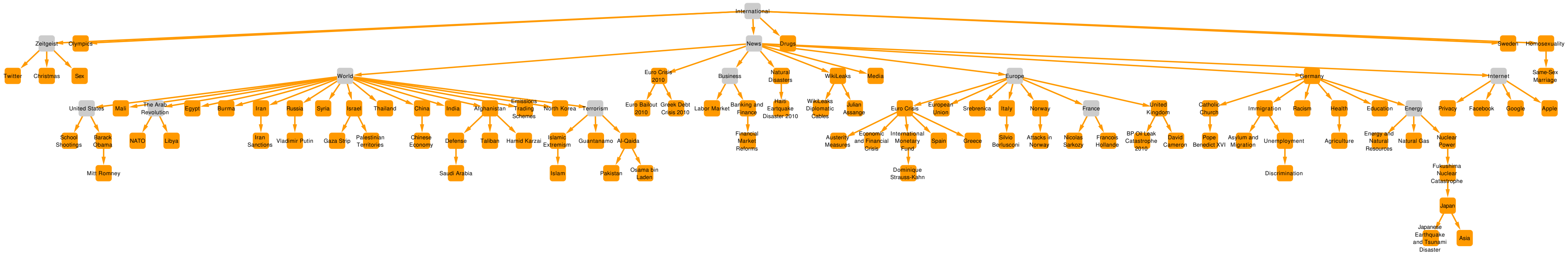

Supplementary Figure S9: Spiegel, reduced for Australian. Use Ctrl-F to search for specific tags.

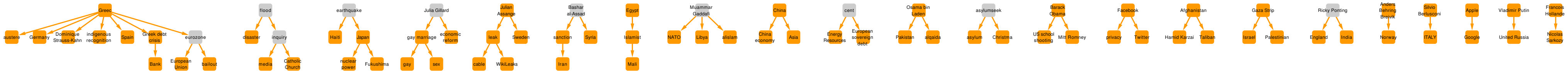

Supplementary Figure S10: Australian, reduced for Spiegel. Use Ctrl-F to search for specific tags.

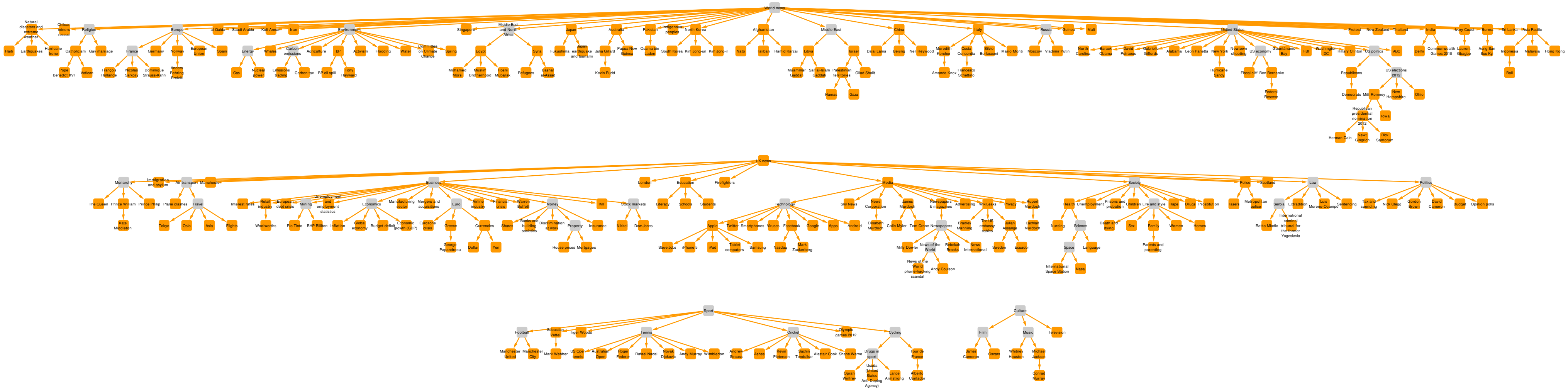

Supplementary Figure S11: Guardian, reduced for Australian. Use Ctrl-F to search for specific tags.

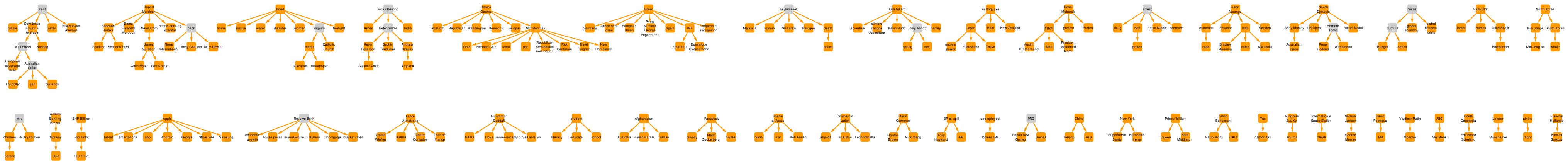

Supplementary Figure S12: Australian, reduced for Guardian. Use Ctrl-F to search for specific tags.

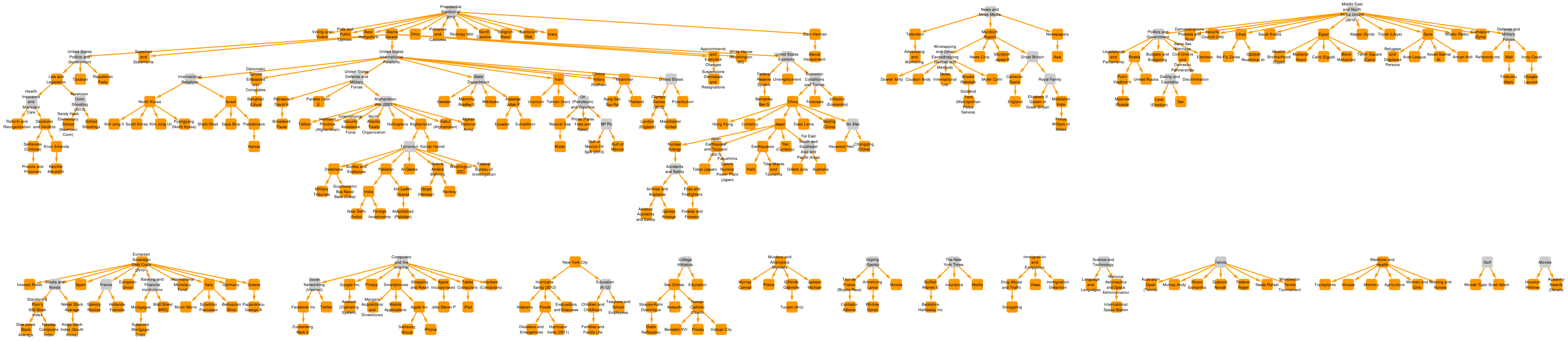

Supplementary Figure S13: New York Times, reduced for Australian. Use Ctrl-F to search for specific tags.

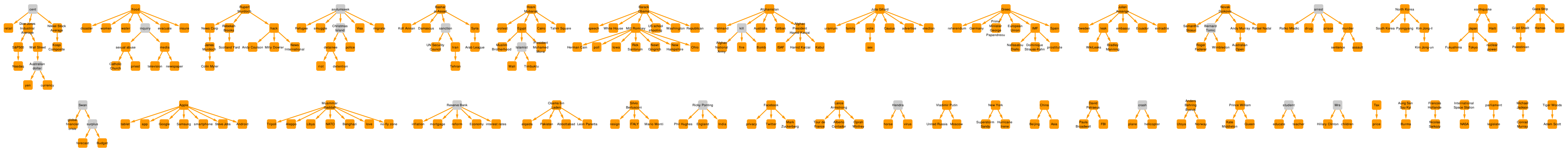

Supplementary Figure S14: Australian, reduced for New York Times. Use Ctrl-F to search for specific tags.

Frequency correlations with parent coappearances

A further property of the datasets is the sensitivity of the strength of the DAGs' parent-child relationships to the frequency of the involved tags. It is quite easy to build a tagged dataset in which infrequent tags appear almost always with their parents, and frequent tags do that on a much smaller ratio of their appearances. As a test, we measure the correlation between the frequency of the children tags and their ratio of coappearances with their parents compared to all of their appearances. Supplementary Table S1 shows while the sign of the correlation is always negative, corresponding to proportionally less coappearances for more frequent tags, the Spiegel and the Guardian have much weaker such correlations that the New York Times and the Australian, suggesting it is easier to find parent-child relationships of the frequent tags for the former two datasets.

Supplementary Table S1: Correlation strength between tag frequency and coappearances with parent over all appearances. The second column corresponds to Spearman's rank correlation, while the third to the usual linear Pearson correlation.

dataset	C_S	C_P
Spiegel	-0.33	-0.07
Guardian	-0.37	-0.06
New York Times	-0.66	-0.36
Australian	-0.68	-0.38

References

[1] Tibély, G., Pollner, P., Vicsek T. & Palla, G. Extracting tag hierarchies. *PLoS ONE* **8,** e84133 (2013).

[2] Lambiotte, R. & Rosvall, M. Ranking and clustering of nodes in networks with smart teleportation. *Phys. Rev. E* **85,** 056107 (2012).
